# Liquid Chromatography/Quadrupole Time-of-Flight Mass Spectrometry for Identification of In Vitro and In Vivo Metabolites of Bornyl Gallate in Rats

**DOI:** 10.1155/2013/473649

**Published:** 2013-03-27

**Authors:** Wei Lan, Liujiao Bian, Xinfeng Zhao, Pu Jia, Xue Meng, Yizhen Wu, Shixiang Wang, Sha Liao, Jie Yu, Xiaohui Zheng

**Affiliations:** College of Life Sciences, Northwest University, Xi'an, Shaanxi 710069, China

## Abstract

Bornyl gallate (BG) is a potential drug candidate synthesized by the reaction of two natural products, gallic acid and borneol. Previous studies have strongly suggested that BG is worthy of further investigation due to antioxidant, antiatherosclerosis activities, and obvious activity of stimulating intersegmental vessel growth in zebrafish. This work was designed to elucidate the metabolic profile of BG through analyzing its metabolites in vitro and in vivo by a chromatographic separation coupled with a mass spectrometry. The metabolites of BG were characterized from the rat liver microsome incubation solution, as well as rat urine and plasma after oral administration. Chromatographic separation was performed on an Agilent TC-C_18_ column (250 mm × 4.6 mm, 5 **μ**m) with gradient elution using methanol and water containing 0.2% (V : V) formic acid as the mobile phase. Metabolites identification involved analyzing the retention behaviors, changes of molecular weights and MS/MS fragment patterns of BG and the metabolites. Five compounds were identified as isomers of hydroxylated BG metabolites in vitro. The major metabolites of BG in rat urine and plasma proved to be BG-O-glucuronide and O-methyl BG-O-glucuronide. The proposed method confirmed to be a reliable and sensitive alternative for characterizing metabolic pathways of BG.

## 1. Introduction

Traditional Chinese medicine (TCM), which serves as a resource of bioactive compounds for drug discovery, is attracting increasing global attention [1]. In practice, it is generally prescribed as a combination of several herbal species and/or minerals to improve therapeutic effects. So far, there have been 12,806 medical resources found in China, including 11,145 medicinal plants, 1581 medicinal animals, and 80 medicinal minerals [2]. Such ample Chinese natural medicinal resources provide valuable materials for the discovery and development of new drugs. More importantly, the clinic medicinal experience of more than 2000 years made the TCM-derived active compounds better lead compounds for further chemical improvements.

**Figure 1:**
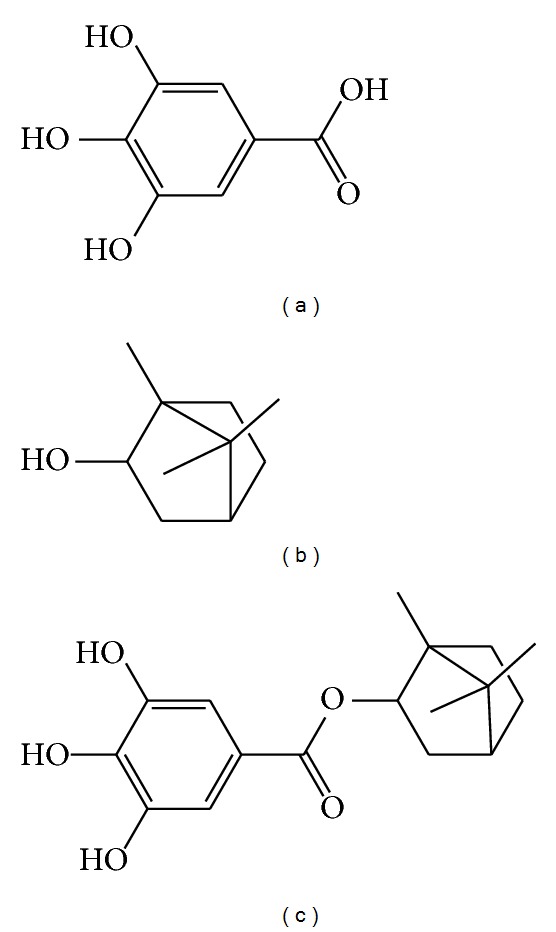
Chemical structures of gallic acid (a), borneol (b), and bornyl gallate (c).

Gallic acid (3,4,5-trihydroxybenzoate) (Figure 1(a)), an endogenous plant phenol, is found abundantly in tea, grapes, different berries, and other fruits as well as wine. It is also isolated from various TCMs such as *Galla Chinensis*, *Choerospondiatis Fructus*, *Radix Paeoniae Rubra*, and many others [3, 4]. Plenty of studies demonstrate that gallic acid has strong antioxidant, anti-inflammatory, and anticancer activities [5, 6]. In particular, the protective effects of gallic acid on cardiovascular diseases have attracted increasing attention in recent studies [7–9]. Borneol (endo-1,7,7-trimethyl-bicyclo[2.2.1]heptan-2-ol) (Figure 1(b)), as an adjuvant drug in many formulae of TCMs, is believed to play assisting roles in facilitating the delivery of principal components, increasing bioavailability, and helping active compounds penetrate the biological barriers [10, 11]. For the purpose of developing new drugs for treatment of cardiovascular diseases, we synthesized bornyl gallate [1,7,7-trimethylbicyclo[2.2.1]heptan-2-yl 3,4,5-trihydroxybenzoate (BG)] (Figure 1(c)) through the dehydration reaction of gallic acid and borneol. Bornyl gallate will not only keep the bioactivities from gallic acid but also get higher penetrability for the existence of borneol moiety. Besides the reported antioxidant activity of bornyl gallate [12], previous studies in our laboratory also showed that BG has obvious activity of stimulating intersegmental vessel growth in zebrafish and potential for antiatherosclerosis by suppressing monocyte activation and foam cell formation [13]. These results strongly suggest that BG is worthy of further investigation.

Metabolite identification is becoming increasingly important in the early stage of drug discovery as a basis for judging whether or not a drug candidate merits further development [14]. Through metabolite identification, we are able to get a quick look at the metabolic fate of a parent drug, determine the major metabolic pathways, and also find whether or not any potentially reactive or toxic metabolites are formed. A great deal of the structural information of metabolites can be obtained using the state-of-art liquid chromatography-mass spectrometry (LC-MS) strategies available now. In several different LC/MS platforms, quadrupole time-of-flight mass spectrometry (Q-TOF/MS) is adopted in this paper because it provides elemental composition from accurate mass measurement and metabolite structures can be proposed with high degrees of certainty without the need of standards for each metabolite [15, 16].

In order to predict the safety and efficacy of BG, it is extremely important to identify its metabolites and thoroughly understand its metabolic fate. Therefore, we firstly analyzed the in vitro metabolites of BG after incubating with rat liver microsome (RLM), subsequently investigated the metabolic profiles of BG in rat plasma and urine, and tentatively identified in vivo metabolites by comparing MS/MS fragment patterns and change of molecular mass with those of the parent drug.

## 2. Experimental

### 2.1. Chemicals and Reagents

Bornyl gallate (purity: >99%, HPLC) was synthesized and identified by ^1^HNMR, IR, and LC/Q-TOF/MS in our laboratory. HPLC grade methanol was purchased from Fisher Chemical Co., Inc. (CA, USA). HPLC grade formic acid was purchased from Kermel Chemical Reagent Co., Ltd. (Tianjin, China). **β**-NADP, D-glucose 6-phosphate and glucose-6-phosphate dehydrogenase were from Sigma-Aldrich Chemical Co. (MO, USA). Water was double distilled in the laboratory. All other reagents used are analytical grade unless stated specially.

### 2.2. Animals

Male Sprague-Dawley rats (200–220 g) were purchased from the Laboratory Animal Research Center of Xi'an Jiaotong University (Shaanxi, China). The rats were kept in metabolic cages in a breeding room with temperature at 23 ± 2°C, relative humidity of 55 ± 10%, and a 12 h light-dark cycle. They were fed with standard laboratory food and water for at least 3 days before experimentation. All experiments on animals were performed in accordance with the university guideline and approved by the Ethical Committee for Animal Care and Use of Northwest University, China.

### 2.3. In Vitro Sample Preparation

Rats were starved overnight before sacrificed. Minced livers were homogenized in 4 × volume of microsome buffer (pH 7.4) containing 0.1 M potassium phosphate, 10% sucrose, 0.1 mM EDTA, 2 mM dithiothreitol, and 1 mM phenylmethylsulfonyl fluoride prior to be centrifuged at 9,000 ×g for 30 min (4°C). Then, the supernatant was further centrifuged at 100,000 ×g for 60 min (4°C). The resulted microsomal pellet was resuspended in fresh microsome buffer and centrifuged again (100,000 ×g, 60 min, 4°C). The collected RLM was dissolved in fresh microsome buffer and immediately stored at −80°C until next use.

Incubations were performed at 37°C in a system containing 3.06 mg/L BG, 0.5 mg/mL rat liver microsomal protein in 0.5 mL of 0.1 mol/L phosphate buffer (pH 7.4). After preincubation at 37°C for 3 min, the reaction between BG and RLM was started by adding a NADPH-regenerating buffer consisting of 1.3 mM **β**-NADP, 3.3 mM D-glucose-6-phosphate, 3.3 mM MgCl_2_, and 0.4 U/mL glucose-6-phosphate dehydrogenase. Incubation was terminated after 30 min by adding ice-cold acetonitrile. Then, 1.0 mL ethyl acetate was used to extract the reacting products from the incubating solution. After being swirled for 60 s and centrifuged at 10,000 ×g for 10 min at 4°C, the supernatant was transferred into a clean tube and evaporated under a gentle stream of nitrogen. The residue was dissolved in 50 *μ*L of 50% methanol water and centrifuged at 10,000 ×g for 10 min. A 25 *μ*L aliquot of the supernatant was injected for LC/MS analysis. Negative control samples were prepared in the same way besides the step of adding ice-cold acetonitrile before the NADPH-regenerating buffer.

### 2.4. In Vivo Sample Preparation

Five male Sprague-Dawley rats were starved overnight with free access to water. Blank blood and urine from each rat were collected prior to dosing. The BG was suspended in 0.5% CMC-Na and orally administered to rats at a dose of 50 mg/kg. 0.5 mL blood samples were collected through ophthalmic veins using heparinized tubes under anesthesia at 1 h after dose. Plasma was prepared by centrifuging the blood for 10 min at 8,000 ×g. Urine samples were collected individually during the time period 0–12 h. The plasma and urine samples were stored at −20°C before further preparation.

All the samples were thawed at room temperature. 0.2 mL acetonitrile containing 0.5% formic acid was added into 0.2 mL plasma or urine. The mixture was thoroughly swirled for 2 min and then centrifuged at 8,000 ×g for 10 min to remove protein in the sample. The supernatant was filtered by 0.45 *μ*m membrane and a 25 *μ*L aliquot was injected into LC/MS system for on-line analysis.

### 2.5. HPLC/Q-TOF/MS Conditions

Chromatographic experiments were performed on an Agilent 1200 series HPLC system, equipped with binary pump, autosampler, on-line degasser and automatic thermostatic column oven (CA, USA). HPLC separation was achieved on an Agilent TC-C_18_ column (4.6 mm × 250 mm, 5 *μ*m) protected by an Agilent TC-C_18_ guard column (4.6 mm × 12.5 mm, 5 *μ*m) with the column temperature set at 30°C. The mobile phase consisted of water containing 0.2% (V:V) formic acid (pH 2.2) (A) and methanol (B) using a gradient elution of 10% B at 0–15 min, 10% to 85% B at 15–60 min, and 95% B at 60–65 min. The flow rate was 0.7 mL/min with an injection volume of 25 *μ*L.

To identify the metabolites in the elution, the HPLC system was coupled online to an Agilent 6500 series quadrupole-time of flight mass spectrometer (Q-TOF/MS), equipped with a dual electrospray ionization source (Dual-ESI) (CA, USA). The LC effluent was introduced into the ESI source in a postcolumn splitting ratio of 3 : 1. Mass spectra were acquired in negative ion mode with the mass range set at m/z 100–1000. The conditions used for the ESI source included a capillary voltage of 4000 V, a drying gas temperature of 350°C, a drying gas flow of 10 L/min, and a nebulizer pressure of 35 psi as well as a fragmentor voltage of 125 V. Internal reference masses in negative mode were set at m/z 112.9855 and 966.0007. MassHunter Workstation software from Agilent Technologies (CA, USA) was used for data acquisition and processing in full-scan and targeted MS/MS modes.

## 3. Results and Discussion

### 3.1. LC-MS Analysis of Bornyl Gallate

**Figure 2:**
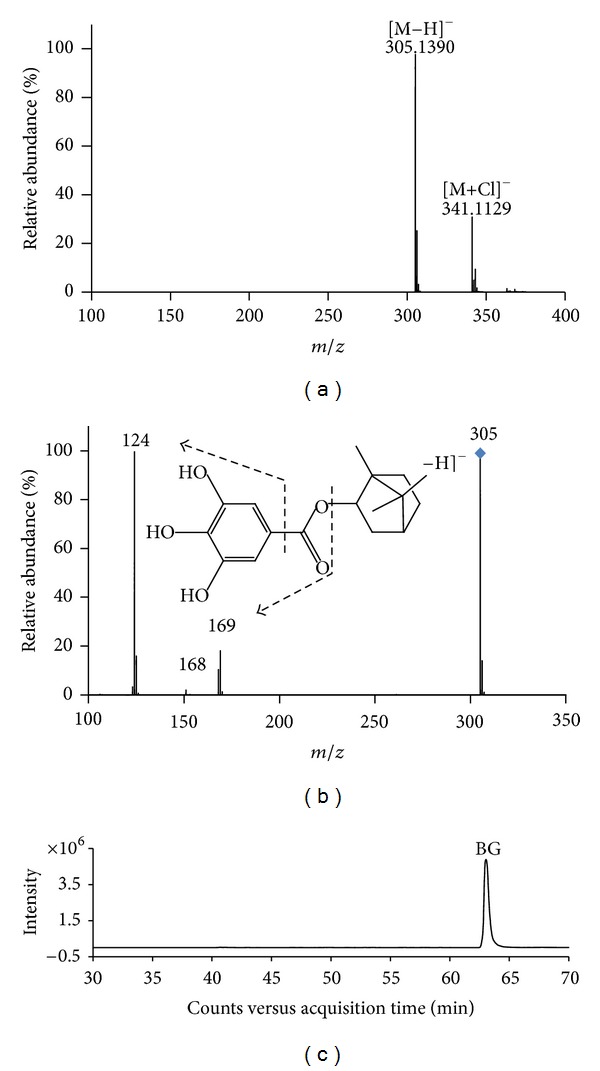
MS, MS/MS spectrum, and total ion chromatogram (TIC) of reference BG (a) MS spectrum; (b) MS/MS spectrum, and the predominant fragmentation pattern; (c) TIC of BG. The chromatographic separation is performed on an Agilent TC-C_18_ column (250 mm × 4.6 mm, 5 *μ*m) with gradient elution using methanol and water containing 0.2% (V : V) formic acid as the mobile phase. Under the proposed condition, the retention time of BG was determined to be 63.0 min.

The HPLC-MS conditions were optimized to provide a full overview of the pattern of the metabolites in rat plasma and urine after oral administration of BG. Ionization of the parent drug BG was much better in the negative mode than that in the positive mode, and the difference between the chromatograms of blank samples and those of samples after oral dosing was more noticeable in the negative mode. Therefore, metabolite identification was performed in the negative ionization mode. Under the proposed condition, the retention time of BG was determined to be 63.0 min (Figure 2(c)). Full-scan analysis generated a mass spectrum of BG (Figure 2(a)) attributing to a negative deprotonated ion [M−H]^−^ at m/z 305.1390 and an adduct ion [M+Cl]^−^ at m/z 341.1129. Precursor ion at m/z 305 gave daughter ions at m/z 169, m/z 168, m/z 124, and m/z 125 (Figure 2(b)). Directly loss of borneol moiety produced the ion at m/z 168. The daughter ion at m/z 169 was generated due to the McLafferty rearrangement cleavage based on gallic acid group. Ions at m/z 124 and m/z 125 attributed to the loss of CO_2_ from the above two ions, respectively. The four ions were subsequently used as diagnostic product ions to ensure whether an unknown metabolite is formed from BG through comparing difference of mass lost between BG and the metabolite.

### 3.2. Identification of In Vitro Metabolites in RLM

**Figure 3:**
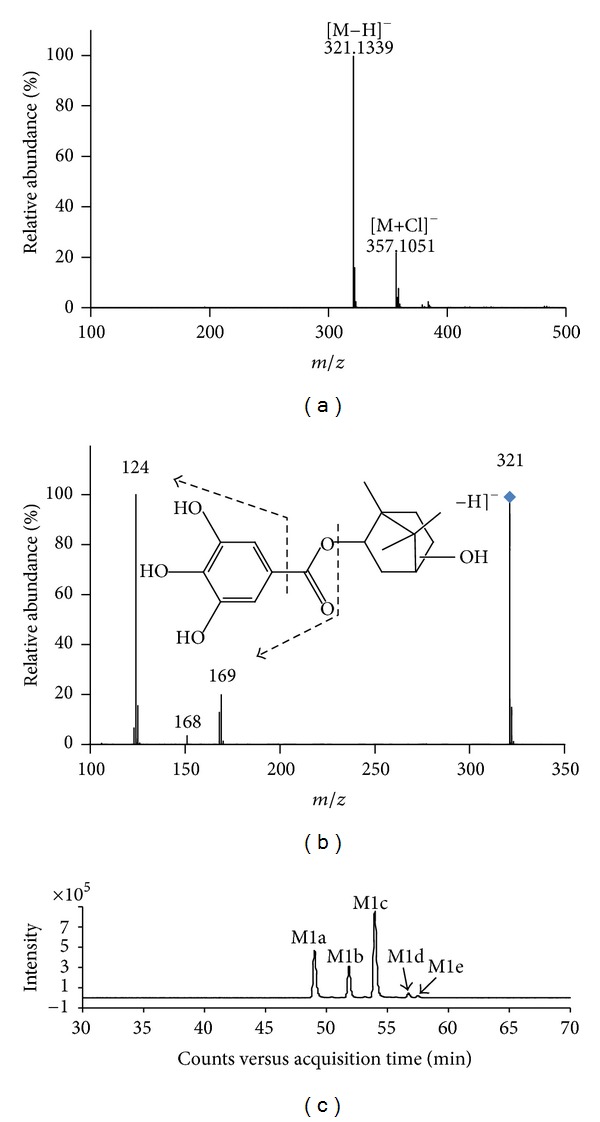
Representative MS, MS/MS spectrum, and extracted ion chromatogram (EIC) of in vitro metabolites of BG in rat liver microsomes (RLM). Incubations were performed at 37°C for 30 min in a system containing 3.06 mg/L BG and 0.5 mg/mL liver microsomal protein in 0.5 mL of 0.1 mol/L phosphate buffer (pH 7.4). (a) MS spectrum; (b) MS/MS spectrum and the predominant fragmentation pattern of M1a are shown as the representative spectra of five metabolites, which exhibited almost identical spectra; (c) EIC of m/z 321.1339 representing 5 isomers of in vitro metabolites with their RTs at 49.0, 51.8, 54.0, 56.8, and 57.5 min, respectively.

Compared with the negative control sample, five new compounds (M1a, M1b, M1c, M1d, and M1e) and the parent drug BG were detected in RLM incubation solution. The full-scan mass spectrum analysis revealed that the five compounds had molecular ions [M−H]^−^ at m/z 321.1339, 321.1341, 321.1340, 321.1338, and 321.1335, respectively. All ions had identical calculated formula of C_17_H_22_O_6_ (mass_calc._ = 321.1344, error <2.7 ppm), representing the notable difference of a single oxygen atom from BG. This interesting result indicated that the new compounds found in vitro should be isomers of monohydroxylated BG. Further MS/MS spectra of each compound at m/z 321 provided same product ions at m/z 169, 168, 124 and 125, which remains same as the MS^2^ fragment ions of the parent drug. The full-scan mass spectrum, MS/MS spectrum, and the predominant fragmentation pattern of M1a are shown in Figure 3(a) and Figure 3(b) as the representative spectra of five metabolites, which exhibited almost identical spectrum. It is accordingly believed that the compounds are metabolites of BG. The data above strongly suggests that hydroxylation of BG took place in borneol moiety while the gallic acid moiety was not modified by enzymes present in RLMs.

The retention times of the five metabolites were determined to be 49.0, 51.8, 54.0, 56.8, and 57.5 min from the extracted ion chromatogram of m/z 321.1339 (Figure 3(c)). It is found that the retention times were all shorter than those of BG, suggesting stronger polarity of the metabolite than BG. For the isomers themselves, the polarities decreased as the order of M1a, M1b, M1c, M1d, and M1e. Among the isomers, M1a, M1b, and M1c should be main metabolites of hydroxylation based on the ion abundance.

### 3.3. Analysis of In Vivo Metabolites of BG

Drug metabolism involves chemical conversion to reduce pharmacological activity of a drug candidate and to facilitate its elimination from the body. Metabolic processes can also produce metabolites that are more pharmacologically active. These metabolic reactions are generally divided into two cases called phase I and phase II reactions. According to the rules of metabolic reactions and the results from in vitro experiments, we predict that the probable metabolic reactions of BG involve in phase I reactions including hydroxylation and hydrolysis as well as phase II reactions such as glucuronidation, O-methylation, and sulfation plus acetylation. The possible structures of metabolites in vivo have been analyzed based on the above theory. Additionally, the calculated molecular formulas and mass values (m/z) of corresponding metabolites have been generated by a tool of Mass Calculator in software Qualitative Analysis B.04.00 (MassHunter Workstation, Agilent, USA).

#### 3.3.1. Metabolic Profile of BG

**Figure 4:**
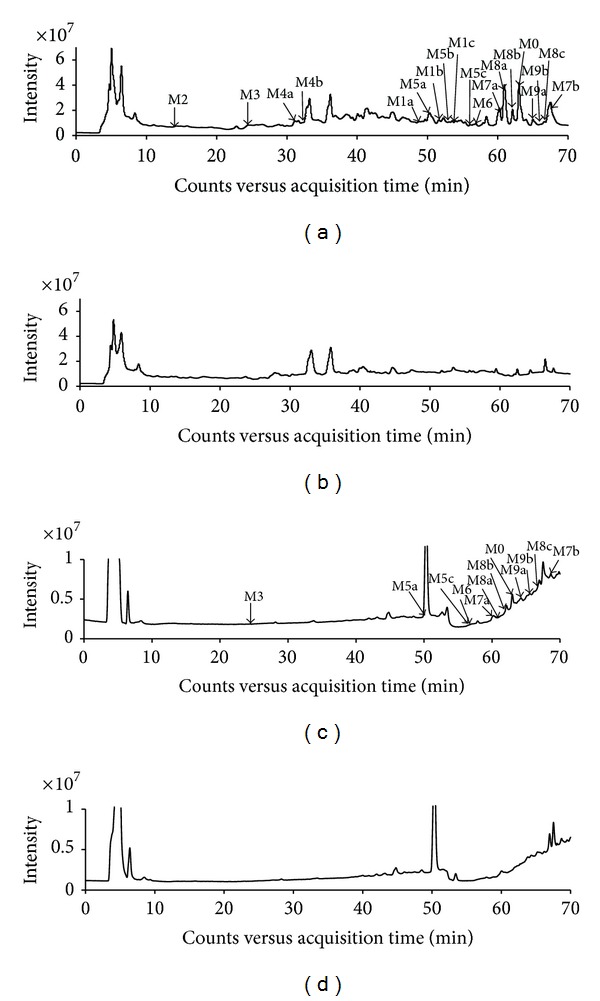
Total ion chromatograms (TICs) of rat urine and plasma samples by HPLC/Q-TOF/MS. (a) TIC of the urine sample after oral administration at a single dose of 50 mg/kg BG; (b) TIC of blank urine; (c) TIC of the plasma sample after oral administration at a single dose of 50 mg/kg BG; (d) TIC of blank plasma.

**Figure 5:**
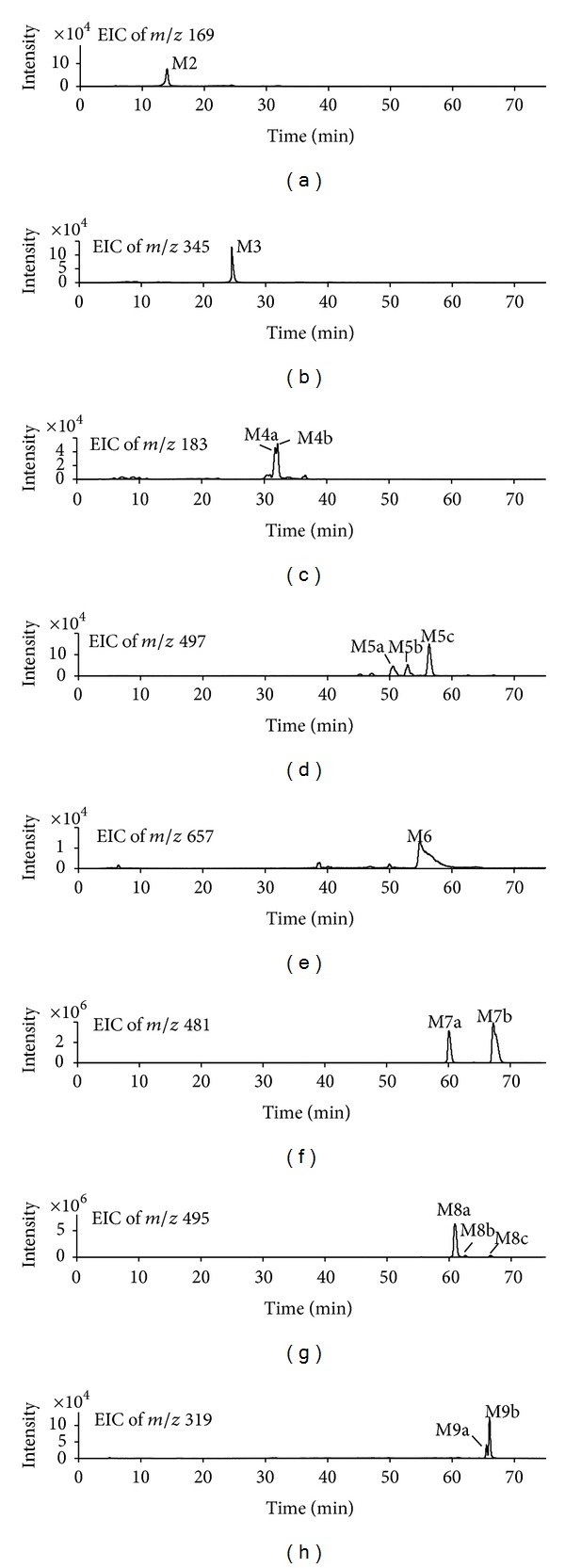
The extracted ion chromatograms (EICs) of in vivo metabolites M2 to M9 of BG in rat urine samples.

**Figure 6:**
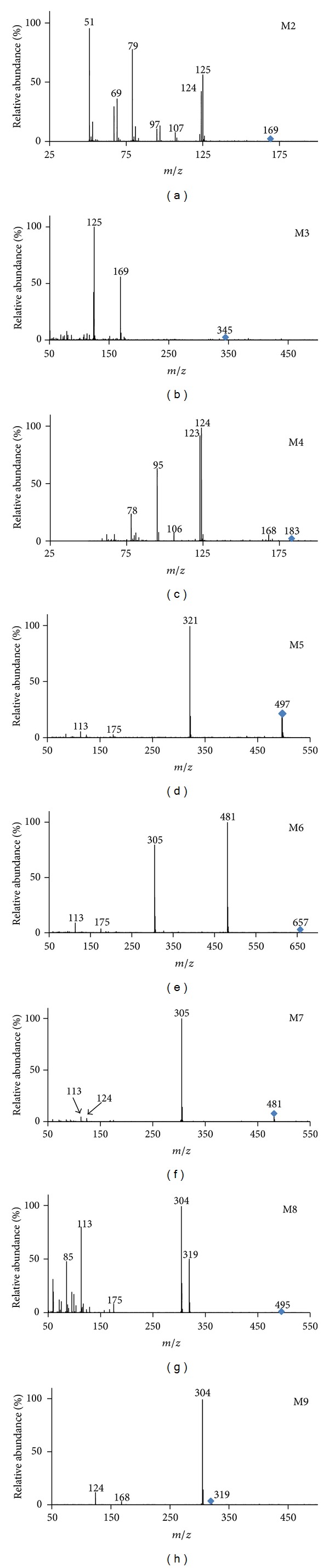
Representative MS/MS spectra of the in vivo metabolites M2 to M9 of BG in rats.

The total ion chromatograms (TICs) of blank samples and samples after oral dosing in negative ion mode are shown in Figure 4. In order to increase sensitivity and to eliminate the endogenous interferences from complex biological matrices, the extracted ion chromatograms (EICs) were used to confirm the existence of potential metabolites by comparing the EICs of the samples after oral dosing with those of blank samples (Figure 5). As a result, the parent drug BG (M0) was detected in both urine and plasma. 9 kinds of potential metabolites were detected in urine. These metabolites have deprotonated ions [M−H]^−^ of m/z 321 (M1), 169 (M2), 345 (M3), 183 (M4), 497 (M5), 657 (M6), 481 (M7), 495 (M8), and 319 (M9), respectively. Among them, M3, M5, M6, M7, M8, and M9 were detected in plasma after oral administration. Most of the potential metabolites, except M1, M2, M3, and M6, have two or three isomers due to the existence of 3 phenolic hydroxyl groups in the molecular structure, which have the properties of undergoing phase II conjugation reactions. The lowercase letters in alphabetical order were used to represent the elution orders of isomers of corresponding metabolites. Furthermore, the structures of metabolites were elucidated based on the targeted MS/MS spectra (shown in Figure 6). However, their exact conjugation sites could not be identified in this work.  Table 1 showed the retention time (RT), measured mass, their calculated formula by elemental compositions, the mass error between the calculated and measured values, MS/MS fragment ions, and metabolic pathway for each metabolite as well as relative peak ratio [17] which could be useful to estimate the main metabolites. 

#### 3.3.2. Identification and Structure Elucidation of In Vivo Metabolites

In the EIC of m/z 321 (M1), three chromatographic peaks of isomers (RT at 49.0, 51.8, and 53.9 min) were observed in urine. M1 showed 16 mass units higher than those of parent drug M0, indicating that they were the monohydroxylated BG. Their retention times and MS^2^ fragments were the same as those of M1a, M1b, and M1c in RLM incubation solution in section of in vitro investigation. 

M2 was eluted at 13.9 min with the [M−H]^−^ ion at m/z 169.0145 (calculated formula = C_7_H_6_O_5_). The MS/MS spectrum of m/z 169 gave abundant daughter ion at m/z 125, which were produced by the loss of CO_2_ (−44 Da) from precursor ion. Moreover, either retention time or fragment ion of M2 was identical as that of gallic acid by comparing with an authentic standard. Thus, M2 was identified as gallic acid, the hydrolysis product of BG. 

M3 and M4 were tentatively assigned as metabolites originating from gallic acid. M3 gave a deprotonated molecule [M−H]^−^ at m/z 345.0469 (calculated formula = C_13_H_14_O_11_). Its MS/MS fragmentation was predominated by the elimination of glucuronide moiety (176 Da) to give product ion at m/z 169. M3 was identified as gallic acid-O-glucuronide. Although baseline separation was not achieved, two peaks were obviously observed in urine containing BG in the EIC of M4. The two peaks represented two isomers M4a and M4b, giving deprotonated molecular ions at m/z 183.0302 and 183.0304 (calculated formula = C_8_H_8_O_5_) and daughter ions at m/z 168 [M−H-CH_3_]^−^ and m/z 124 [M−H-CH_3_-COO]^−^. The two isomers were considered to be O-methylation products of gallic acid, 4-O-methylgallic acid, or 3-O-methylgallic acid. Based on the previous studies on its metabolic fate, gallic acid will be metabolized through decarboxylation, O-methylation, sulfation, and glucuronidation reactions in rats [18, 19]. In our work, the gallic acid, which was produced by the hydrolysis of BG, proved to undergo further metabolic reactions including O-methylation and glucuronidation. The decarboxylation and sulfation metabolites were not observed, probably because of their low concentration.

Two chromatographic peaks with their RTs at 65.5 (M9a) and 66.0 (M9b) min were detected in the EIC of m/z 319.1556. The MS/MS spectra of m/z 319 showed M9 produced fragment ion at m/z 304 [M−H-CH_3_]^−^, indicating that the metabolites M9a and M9b should be O-methyl BG, the O-methylation products of M0.

All the MS/MS fragmentation patterns of M5, M6, M7, and M8 presented neutral loss of 176 Da from precursor ions. Furthermore, the characteristic ions of glucuronide at m/z 175 and m/z 113 were observed in each of their MS/MS spectra, which confirmed the presence of glucuronide according to the reported literature [20, 21]. M5a, M5b, and M5c gave deprotonated ions [M−H]^−^ at m/z 497.1674, 497.1681, and 497.1678, corresponding to a molecular formula of C_23_H_30_O_12_. Their retention times were determined to be 50.5, 52.9 and 56.3 min, respectively. The targeted MS/MS spectra yielded a daughter ion at m/z 321. A loss of 176 Da to precursor ions is easily calculated for identifying M5a, M5b, and M5c as isomers of monohydroxylated BG-O-glucuronide, the glucuronidation products of M1. M6 presented a deprotonated ion [M−H]^−^ at m/z 657.2043 and the daughter ions at m/z 481 and m/z 305. The two daughter ions denote the loss of 176 Da and 2 × 176 Da to the precursor ion at m/z 657, which paved the way to recognize M6 as BG-di-O-glucuronide. M7 had two isomers and showed the [M−H]^−^ ions at m/z 481.1722 and 481.1728. In their MS/MS spectra, M7 further lose a glucuronic acid moiety (176 Da) to produce ions at m/z 305, indicating that M7a and M7b were BG-O-glucuronide, the glucuronide conjugates of M0. M8 had three isomers (M8a, M8b, and M8c), giving the deprotonated ions [M−H]^−^ at m/z 495.1883, 495.1879, and 495.1877. The fragment ions of them were all investigated at m/z 319 and 304, indicating a neutral loss of 176 Da and the additional loss of CH_3_ (−15 Da). M8a, M8b, and M8c thus were characterized as glucuronide conjugates of M9, O-methyl-BG-O-glucuronide.

### 3.4. Metabolic Pathway of BG

**Figure 7:**
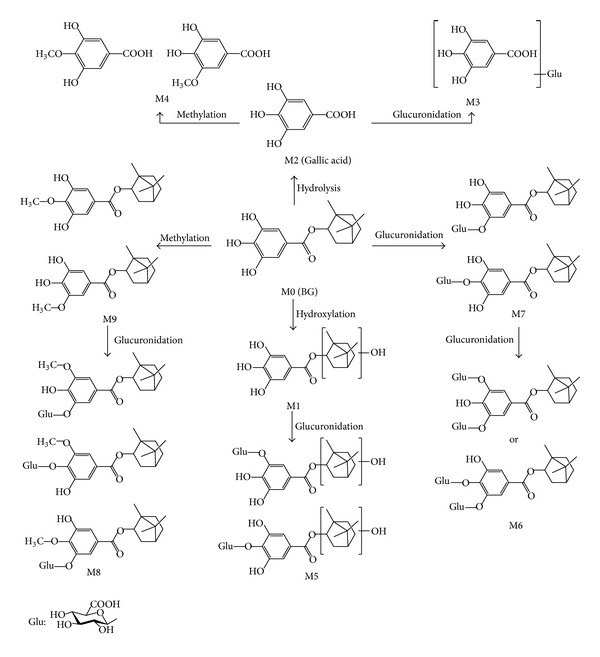
Proposed metabolic pathways of BG in rats.

Based on the above discussion, the proposed metabolic pathways of BG in rats were presented in Figure 7. In the metabolic profile, BG (M0) was detected in plasma and excreted through urine. BG-O-glucuronide (M7) and O-methyl BG-O-glucuronide (M8) were the most abundant metabolites in vivo according to the relative peak ratio. The hydroxylation and hydrolysis metabolites in low concentration were observed only in the urine, while their further glucuronide conjugates were detectable in plasma. All of these results indicated that the major metabolites were present as conjugated forms in vivo and the main metabolic pathways of BG were glucuronidation and O-methylation. 

## 4. Conclusion

In this paper, a reliable and sensitive HPLC/Q-TOF/MS method was successfully applied to identify the metabolites of bornyl gallate in vitro and in vivo for the first time. In vitro, BG was metabolized to five isomers of monohydroxylated BG by CYP450 enzymes present in RLM. In vivo, 9 kinds of potential metabolites, altogether 18 compounds including all isomers, were detected and identified. BG is believed to undergo various phases I and II metabolic pathways including hydroxylation, hydrolysis, O-methylation, and glucuronidation, while the conjugation with sulfation or acetylation was not detected. We also proved that BG mainly became products of glucuronidation and O-methylation in vivo, which were identified as BG-O-glucuronide (M7) and O-methyl BG-O-glucuronide (M8).

## Figures and Tables

**Table 1:** Retention time (RT), measured mass, calculated formula by elemental compositions, the mass error between the calculated and measured values, MS/MS fragment ions, and metabolic pathway as well as relative peak ratio for each metabolite of BG in rat plasma and urine after oral administration.

Compound	RT (min)	Measured mass (*m*/*z*)	Mass error (ppm)	Relative peak ratio^a^ in urine	Relative peak ratio in plasma	Formula	MS/MS fragment ions	Metabolic pathway
M0	63.0	305.1400	−1.8	100	100	C_17_H_22_O_5_	169, 168, 125, 124	Parent drug
M1a	49.0	321.1350	−2.0	0.68	*∖* ^ b^	C_17_H_22_O_6_	169, 168, 125, 124	Hydroxylation
M1b	51.8	321.1348	−1.4	0.53	*∖*			
M1c	53.9	321.1349	−1.7	0.41	*∖*			
M2	13.9	169.0145	−1.5	0.69	*∖*	C_7_H_6_O_5_	125, 124, 79, 51	Hydrolysis
M3	24.4	345.0469	−1.6	3.66	1.38	C_13_H_14_O_11_	169, 125, 124	Hydrolysis + glucuronidation
M4a	31.8	183.0302	−1.7	0.38	*∖*	C_8_H_8_O_5_	168, 124, 123, 95, 78	Hydrolysis + O-methylation
M4b	32.1	183.0304	−2.8	0.32	*∖*			
M5a	50.5	497.1674	−1.9	0.99	2.86	C_23_H_30_O_12_	321, 175, 113	Hydroxylation + glucuronidation
M5b	52.9	497.1681	−3.3	0.90	*∖*			
M5c	56.3	497.1678	−2.7	2.44	2.36			
M6	56.4	657.2043	−1.0	1.08	13.6	C_29_H_38_O_17_	481, 305, 175, 113	Di-glucuronidation
M7a	60.1	481.1722	−1.4	24.64	105.24	C_23_H_30_O_11_	305, 124, 113	glucuronidation
M7b	67.5	481.1728	−2.6	50.96	916.65			
M8a	60.9	495.1883	−2.3	95.36	31.7	C_24_H_32_O_11_	319, 304, 175, 113	O-methylation + glucuronidation
M8b	62.6	495.1879	−1.4	3.66	16.95			
M8c	66.7	495.1877	−1.1	2.68	2.01			
M9a	65.5	319.1556	−1.6	0.39	3.09	C_18_H_24_O_5_	304, 168, 124	O-methylation
M9b	66.0	319.1560	−2.8	1.31	0.86			

^a^Relative peak ratio was calculated on the basis of EIC as follows: (relative peak ratio) = (the peak area of metabolite)/(the peak area of parent, M0) × 100.

^b^ *∖*: undetected.

## Abbreviations

TCM: Traditional Chinese medicine
BG: Bornyl gallate
RLM: Rat liver microsome
HPLC/Q-TOF/MS: High performance liquid chromatography/quadrupole time-of-flight mass spectrometry
ESI: Electrospray ionization
TIC: Total ion chromatogram
EIC: Extracted ion chromatograms
RT: Retention time.
